# Primary multifocal osseous Hodgkin's lymphoma

**DOI:** 10.1186/1477-7819-6-34

**Published:** 2008-03-17

**Authors:** Clare R Langley, Simon JW Garrett, Jill Urand, Janice Kohler, Nick MP Clarke

**Affiliations:** 1 Orthopaedic Department, Basingstoke and North Hampshire Foundation Trust, Aldermaston Road, Basingstoke, Hampshire, RG24 9NA, UK; 2 Royal Bournemouth Hospital, Castle Lane East, Bournemouth, Dorset, BH7 7DW, UK; 3 Southampton University Hospitals NHS Trust, Tremona Road, Southampton, SO16 6YD, UK

## Abstract

**Background:**

Hodgkin's disease (HD) most commonly presents with progressive painless enlargement of peripheral lymph nodes, especially around the cervical region. A few children have systemic symptoms and weight loss. At the time of diagnosis, osseous involvement is uncommon

**Case presentation:**

A case is described of Primary Multifocal Osseous Hodgkin's Lymphoma in a seven-year-old boy. He presented with a painful swelling in the sternum, and further investigations revealed deposits in his L1 vertebra, the left sacro-iliac joint and the right acetabulum.

**Conclusion:**

The clinical, radiological and histological features of this disease can mimic other medical conditions, including Tuberculosis, making the diagnosis difficult and often leading to delays in treatment. This is a very rare condition and we believe this to be the youngest reported case in the literature.

## Background

Hodgkin's disease (HD) most commonly presents with progressive painless enlargement of peripheral lymph nodes, especially around the cervical region. A few children have systemic symptoms and weight loss. At the time of diagnosis, osseous involvement is uncommon and even in the late stages only 9–35% of cases have any bony involvement [1]. It is therefore extremely rare for patients to present with primary Hodgkin's disease of the bone. If there is no associated extra-osseous involvement, the condition is referred to as primary osseous Hodgkin's lymphoma (POHL). It is termed primary multifocal osseous Hodgkin's lymphoma, if more than one osseous site is involved. The clinical, radiological and histological features of POHL can mimic other medical conditions, thereby making the diagnosis difficult, often leading to delays in treatment

We present a case of a seven-year-old boy diagnosed with primary multifocal osseous Hodgkin's lymphoma. We believe this to be the youngest such reported case and only the third ever reported paediatric case of primary multifocal osseous Hodgkin's lymphoma in the English literature.

## Case presentation

**Figure 1:**
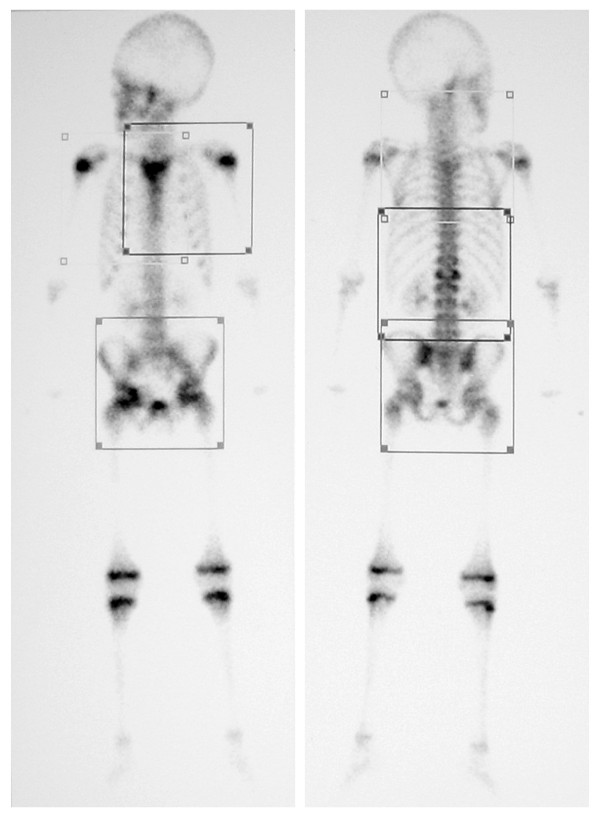
**Bone scan at time of presentation**. Demonstrating increased uptake in the sternum, L1 vertebra, left sacro-iliac joint and right acetabulum.

A seven year old male presented with a painless, firm 3 cm mass overlying his sternum. He was clinically well, apyrexial with no history of weight loss. Initial investigations revealed an elevated CRP (27.5), ESR (90), white cell count (22.3) with a neutrophilia (17.0) and a hypochromic microcytic anaemia (Hb: 9.3). Technecium^99 ^bone scan (Figure 1) revealed increased uptake in the sternum, L1 vertebra, the left sacro-iliac joint and the right acetabulum.

**Figure 2:**
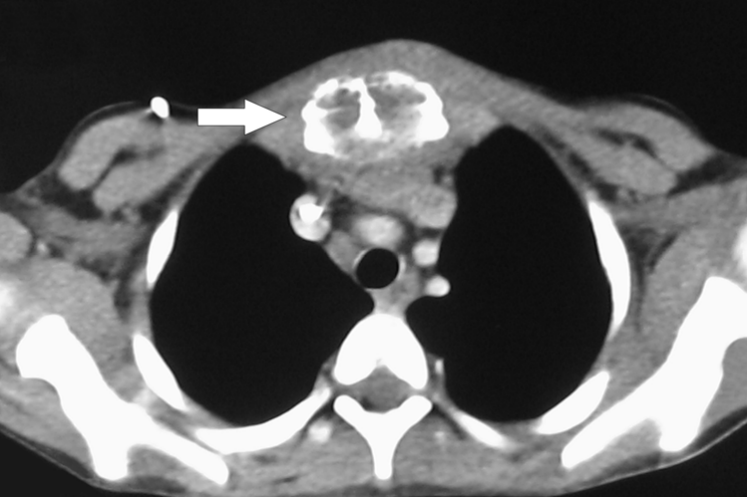
**Axial CT of chest**. Demonstrating the sternal mass (arrow) but no underlying soft tissue involvement.

A CT scan of the chest (Figure 2) and sternum confirmed the presence of a sternal mass with no underlying soft tissue involvement. A fine needle biopsy of the sternal mass showed an inflammatory infiltrate. Bone marrow aspirates and trephine from the sternum showed a reactive marrow with no evidence of malignancy. Both ultrasound of the abdomen and echocardiogram were normal. On the basis of these results a provisional diagnosis of multi-focal osteomyelitis was made and the patient was started on antibiotic treatment (Benzylpenicillin, Flucloxacillin and Fusidic Acid). Despite this treatment his white cell count and inflammatory markers continued to rise.

**Figure 3:**
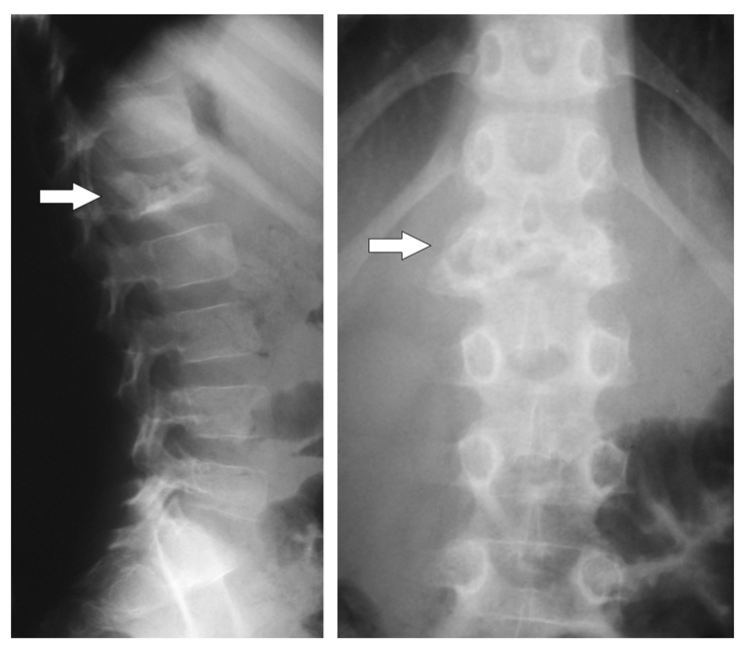
**Plain radiographs (AP and Lateral) of lumbar spine**. Demonstrate destruction of the L1 vertebra (arrow).

**Figure 4:**
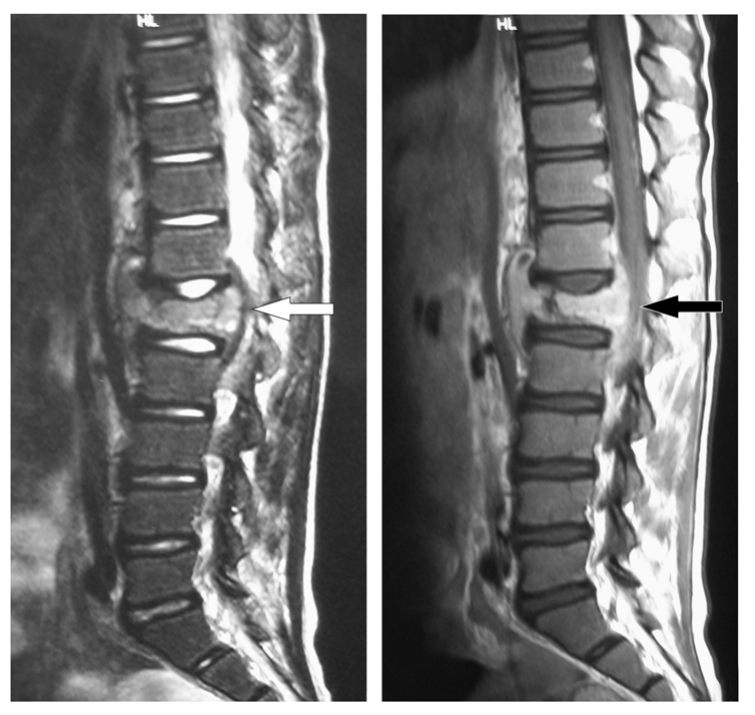
**MRI (T1 and T2 weighted images) of lumbar spine**. Investigation undertaken 6 weeks after presentation showing loss of height of L1 with surrounding soft tissue mass and impingement on the conus.

**Figure 5:**
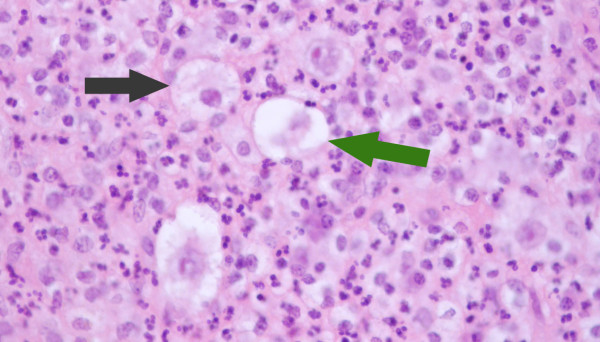
**Histology from sternal aspirate**. Illustrates mixed inflammatory cells, lacunar cells (green arrow) and Hodgkin cells (black arrow).

**Figure 6:**
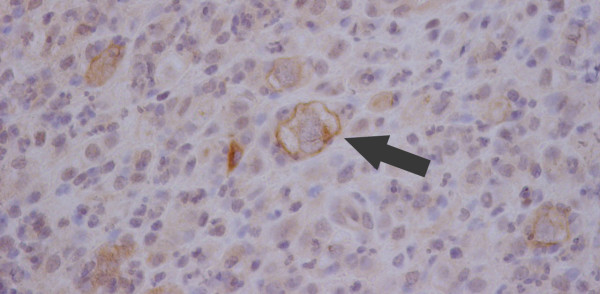
**Histology from sternal aspirate with stain for CD30**. The large cells (arrow) are positive for CD30 found on the surface of Reed-Sternburg cells in Hodgkins Lymphoma.

Five weeks after discharge he represented because of an enlarging sternal mass and the development of back pain with no associated neurology (Figure 3). He remained well with no weight loss or signs and symptoms of systemic disease. An open biopsy of the chest wall mass and a CT guided biopsy of the L1 spinal lesion were performed. These revealed macroscopically caseous material. Mantoux and Heaf tests were negative. An MRI scan of the lumbar spine (Figure 4) showed loss of height of L1 with disease extending bilaterally to the pedicles of T12 and L2. There was a soft tissue mass anterior and posterior to L1 causing spinal stenosis and impingement on the conus. Radiologically this was thought to resemble Potts disease and the macroscopic appearance of the lumbar specimen suggested a diagnosis of tuberculosis. Triple therapy was commenced (Rifampicin, Isoniazid and Pyrazinamide). The specimens were negative for acid fast bacilli, Ziehl-Neelson stain for TB was negative and no organisms were cultured. Subsequent histology from the sternal mass showed Hodgkin's lymphoma (Figure 5). Cells within the specimen were positive for CD30 and CD20 (Figure 6).

**Figure 7:**
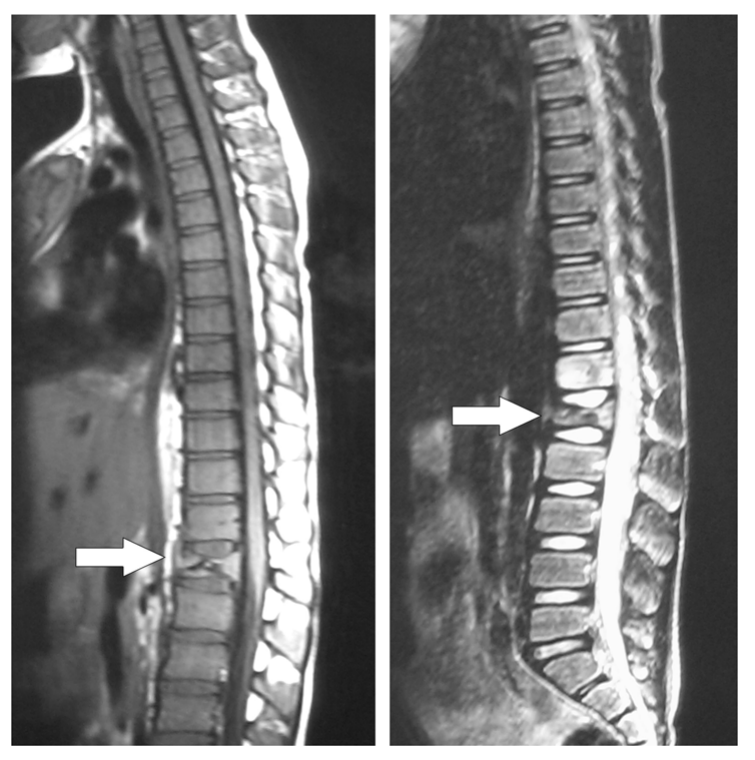
**MRI Thoraco-lumbar spine**. Performed after commencement of chemotherapy regime. This illustrates the decrease in size of L1 lesion and reduction in impingement on the conus.

Treatment with chemotherapy was started following the current UKCCSG (United Kingdom Childrens Cancer Survey Guidelines) for Hodgkin's disease, and antituberculous treatment was stopped. Staging showed no lymphadenopathy in the chest or abdomen. Two weeks after commencement of chemotherapy a dramatic decrease in soft tissue involvement around the spinal cord was seen on a repeat MRI. (Figure 7). The time from presentation to diagnosis was two months.

## Discussion

The incidence of skeletal Hodgkin's disease varies from 9–14% during the course of the disease with up to 30–50% at post mortem [1]. Skeletal involvement may present in four different ways: POHL (either solitary or multifocal); simultaneously in osseous and non-osseous sites; or recurrence of disease at osseous sites. We consider that in order to make a diagnosis of POHL there should not be any signs or symptoms of systemic disease at the time of presentation or at the time of staging. Historically it was felt that primary Hodgkin's of the bone did not occur and that bony involvement was a feature of haematological dissemination of the disease, thereby implying a less favourable prognosis [2]. Granger *et al*., [3] reported a 5 year survival of just 4.2% with 80% of deaths occurring within first 3 years. POHL must therefore be distinguished from systemic HD with diffuse bone marrow involvement (Ann Arbor IV, see table 1), as it appears that POHL may have a better prognosis than systemic Hodgkin's with bony involvement [4]. The most recent case reported in the literature regards a 51 year old female who presented with left hip pain and was subsequently lymphadenopathy in the cervical and inguninal nodes. She was staged as VIB. The question remains as to whether POHL has a better prognosis than HD with bony involvement [5].

**Table 1:** Staging of Lymphoma: Ann Arbor classification

**Stage I **Disease in a single lymph node region
**Stage II **Disease in two or more regions on the same side of the diaphragm
**Stage III **Disease in lymph node regions on both sides of the diaphragm
**Stage IV **Diffuse or disseminated involvement of one or more extralymphatic organs or tissues with or without associated lymph node enlargement.

There are thirty three clearly reported cases of primary osseous lymphoma at either single or multiple sites, in all ages, in the scientific literature since 1927. Table 2 details these 32 cases as well as this current case. It does not include patients who presented with disease at non osseous and osseous sites, or those patients in whom hodgkins disease disseminated to the bone. At least 7 of these cases were reported prior to 1954 when CT, MRI and PET scanning was not available, so it cannot be stated for certain, whether these cases had any evidence of lymphadenopathy within the chest or abdomen. We are uncertain therefore, whether these are true cases of POHL.

**Table 2:** A table of cases in the literature who presented with Hodgkin's lymphoma disease at single or multiple bony sites.

**Year**	**Author and [reference]**	**Age (years)**	**Gender (M/F)**	**Site(s)**	**Therapy**	**Outcome**
1927	Gerbert *et al*. [10]	42	M	T4-T8	XRT	Alive at 10mo LTFU
1936	Gerbert *et al*. [10]	39	F	L humerus	Surgery	DOD 12mo
1943	Gerbert *et al*. [10]	5	F	L scapula	XRT	NED
1958	Gerbert *et al*. [10]	53	M	L Humerus, L Ilium	XRT	DOD at 4mo
1960	Ostrowski *et al*. [6]	73	F	R Femur	XRT	DOD at 4 yrs
1968	Ostrowski *et al*. [6]	34	M	L Humerus	XRT	AWD at 10 yrs
1979	Gerbert *et al*. [10]	25	F	L humerus	XRT	NED 4.5 yrs
1982	Chan *et al*. [1]	12	M	R tibia	XRT & CT	Alive at 66mo
1982	Chan *et al*. [1]	18	M	R ulna, L tibia and fibula	XRT & CT	Alive at 18mo
1982	Chan *et al*. [1]	20	M	T11	XRT & CT	Alive at 15mo
1982	Chan *et al*. [1]	11	F	T8-10, L femur, Pelvis	XRT & CT	Alive at 7mo
1982	Chan *et al*. [1]	68	F	R SI joint	None	Died 2mo
1982	Chan *et al*. [1]	29	M	T10-T12	Surgery	?
1982	Chan *et al*. [1]	45	M	Sternum	Surgery, XRT & CT	Alive at 10mo
1982	Chan *et al*. [1]	21	F	Sternum	Surgery & XRT	Alive at 24mo
1982	Chan *et al*. [1]	41	M	Skull, ischium, L spine	XRT	Died 3mo
1982	Chan *et al*. [1]	17	F	T7 – T8	XRT	Died 24mo
1982	Chan *et al*. [1]	9	M	R tibia	Surgery & XRT	Died 26mo
1982	Chan *et al*. [1]	27	F	R tibia	XRT	Died 3mo
1982	Chan *et al*. [1]	62	M	L humerus	Surgery	Died 1 mo
1982	Chan *et al*. [1]	40	M	T2	Surgery	Died 8mo
1982	Chan *et al*. [1]	24	F	L femur	Surgery & XRT	Died 12mo
1989	Mac Cormick *et al*. [11]	61	M	T spine, R 12^th ^rib, R clavicle	CT	remission
1991	Gross *et al *[7]	17	F	T10, L4, 11^th ^rib, L ilium	CT, XRT, BMT	DOD
1991	Gross *et al *[7]	12	F	T11-12, L3-4, L scapula, L10^th ^rib, L ilium, L acetabulum, L femur	CT	AWD at 2.5 yrs
1993	Borg *et al *[12]	31	M	Sacrum	CT & XRT	NED 5 yrs
1995	Ostrowski *et al*. [6]	61	F	T11	resection & XRT	AWD at 22 mo
1995	Fried *et al*. [13]	21	F	L clavicle	CT	NED 36mo
1995	Gerbert *et al*. [10]	63	M	L femur, R ilium	CT & XRT	NED 6mo
1996	Citow *et al *[8]	54	F	T4, T5	Surgery, CT & XRT	AWD at 36 mo
1999	Gerbert *et al*. [10]	21	M	R femur, R tibia	CT & XRT	NED 48mo
2006	Chandra *et al *[5]	51	F	L ileum	CT & XRT	Alive
2006	Present case	7	M	L1, sternum, Lt SI joint, Rt acetabulum	CT	AWD

CT – chemotherapy

AWD – alive with disease

XRT – Radiotherapy

DOD – died of disease

LTFU – lost to follow up

NED – no evidence of disease

The two cases presented by Ostrowski *et al*., [6] were among 25 patients diagnosed with osseous Hodgkin's disease from a group of over 500 patients known to have had Hodgkin's lymphoma, at the Mayo clinic between 1927 and 1996. Five of the twenty five had POHL; three had disease at a single bony site and two had multifocal bony disease.

Gross *et al*., [7] presented two cases in adolescents (12 years and 17 years) who presented in a very similar pattern to ours. Both presented with back pain and raised inflammatory markers. Investigation revealed widespread osseous involvement. In the case of the 17 year old, treatment was delayed by a misdiagnosis of eosinophilic granuloma. The 12 year old is one of two other paediatric cases of primary multifocal osseous Hodgkin's lymphoma that we have identified. The other case was an 11 year old girl with disease in the thoracic spine, pelvis and left femur [1].

There have been two paediatric cases identified by our literature review of patients with POHL at a single site. A case report by Citow JS *et al*., [8] of a 54 year old female, with back pain and spinal cord compression, thought to be secondary to tuberculosis. Only when antituberculous treatment failed, did re-examination and investigations reveal POHL as the cause.

Radiologically, bony lesions of Hodgkin's disease may be lytic, sclerotic or mixed. One study showed that 75% were lytic, 13.6% mixed and 11.4% mixed [3]. When they involve the vertebral column, the disease can spread from one vertebral body to another across the intra-vertebral disc space and cause destruction of the disc [9].

In all reported cases, the correct diagnosis was only reached after extensive and repeated investigations and review of the histology. The average time to diagnosis from initial presentation was 6–8 months. The most frequent misdiagnosis was osteomyelitis. Our case highlights the difficulties in diagnosing this rare form of Hodgkin's disease.

TB in England has increased by 25 per cent over the last 10 years. Most TB in England occurs among people in inner cities – two in every five cases are in London (see table 3).

**Table 3:** TB in England has increased by 25 per cent over the last 10 years. Most TB in England occurs among people in inner cities – two in every five cases are in London. Tuberculosis in the UK (2004 statistics) from global health facts.

New cases	7101
New case rate (per 100,000)	12
People with TB	5497
TB prevalence (per 100, 000)	9
TB deaths	710
Death rate (per 100, 000)	1

## Conclusion

This case indicates that Primary Multifocal OHL may present in childhood. It demonstrates the difficulties in reaching a definitive diagnosis, and the need to continually evaluate patients and diagnoses, especially when patients fail to respond to initial therapies.

## Competing interests

The author(s) declare that they have no competing interests.

## Authors' contributions

CL, SG, JU and JK all contributed to the literature review. CL,SG and NC have written and revised the manuscripts. All authors read and approved final manuscript for publication.
